# Hand hygiene myths

**DOI:** 10.1186/1753-6561-5-S6-P101

**Published:** 2011-06-29

**Authors:** AJ Stewardson, B Allegranzi, H Sax, C Kilpatrick, D Pittet

**Affiliations:** 1 University of Geneva Hospitals, Geneva, Switzerland; 2 WHO, Geneva, Switzerland

## Introduction / objectives

Professionals promoting hand hygiene (HH) with multimodal strategies featuring the introduction of alcohol-based handrub (ABHR) are confronted by a common set of impeding opinions regarding the indications, safety and efficacy of ABHR. Some of these could be referred to as HH myths.

## Methods

We undertook a review of the literature to assess the currently available evidence regarding five common barriers to the successful implementation ABHR: 1) poor HH before patient contact; 2) the risk of systemic absorption of alcohol; 3) adverse dermatologic effects; 4) risk of *Clostridium difficile* disease; and 5) ABHR as a fire hazard.

## Results

Hand hygiene compliance is usually better after patient contact than before, despite a lack of evidence to suggest this is an effective means to prevent transmission of pathogens. Blood levels of ethanol and acetone after even supra-normal exposure are undetectable or insignificant. Appropriately formulated ABHR products are less likely to result in dermatitis than washing with soap and water. Appropriate implementation of hand hygiene guidelines does not result in *Clostridium difficile* infection incidence. Fire events related to ABHR are extremely rare and almost exclusively associated with inappropriate use.

## Conclusion

Like any medication, ABHRs do have potential adverse effects, but these can be minimised by appropriate usage. Rare or even mythic complications should be weighed realistically against the potential of ABHR to prevent countless healthcare-associated infections each year.

## Disclosure of interest

None declared.

